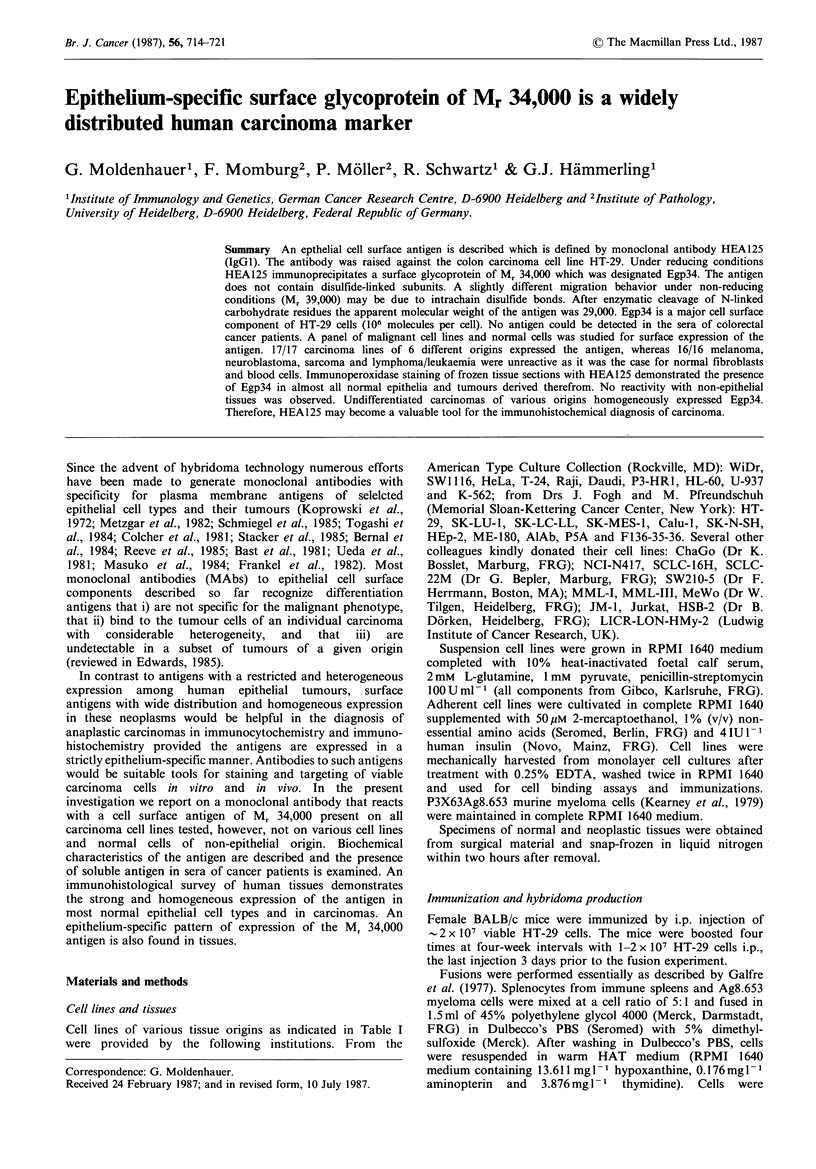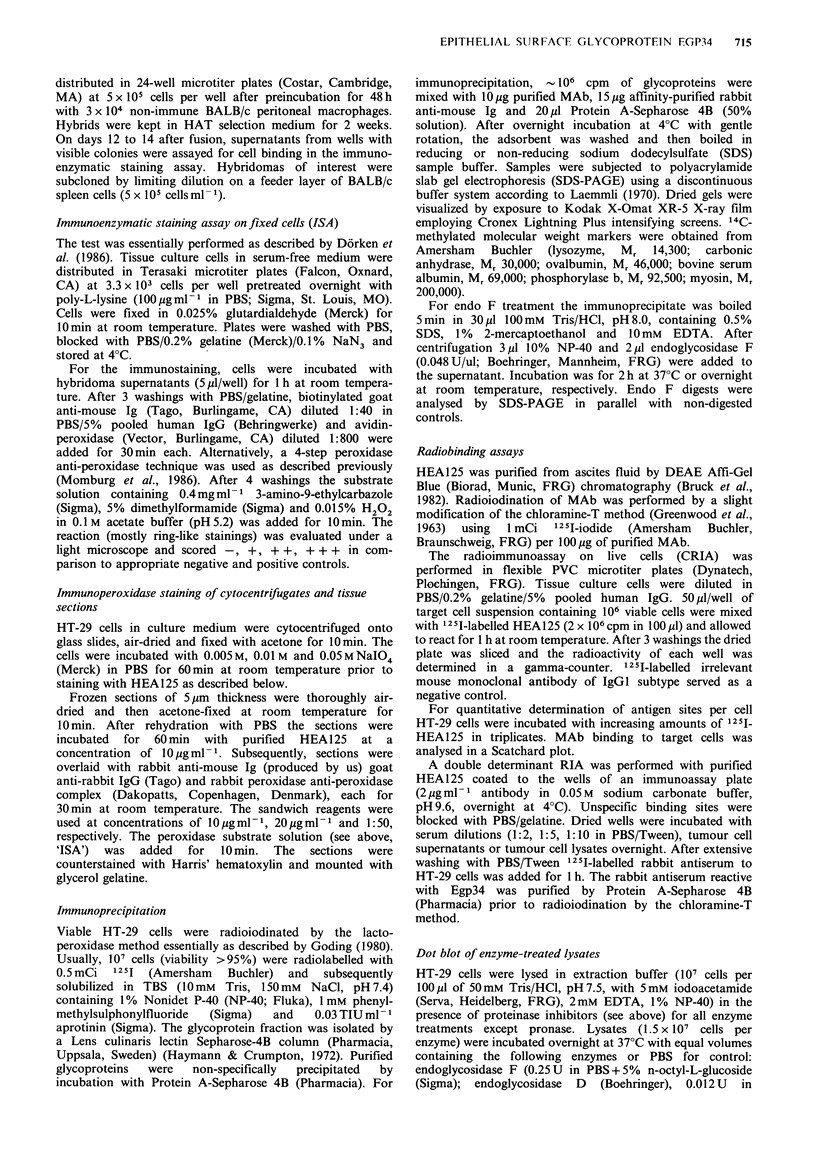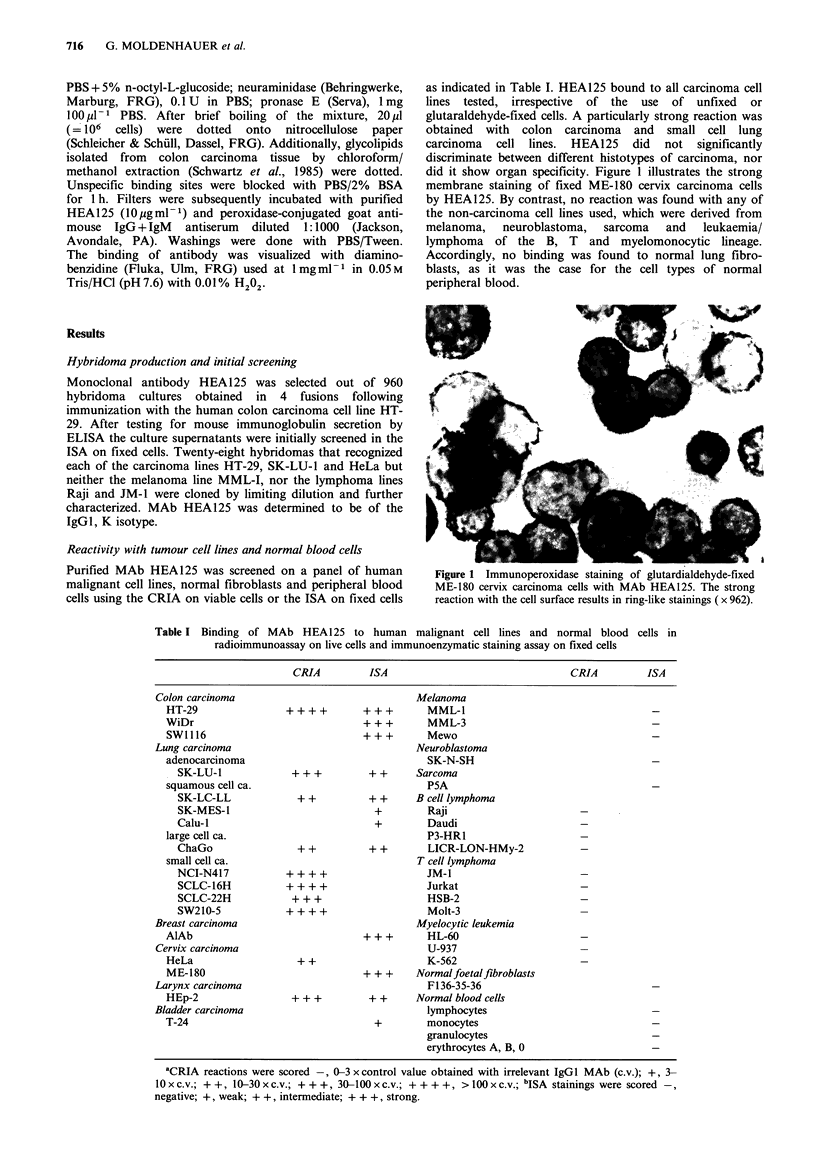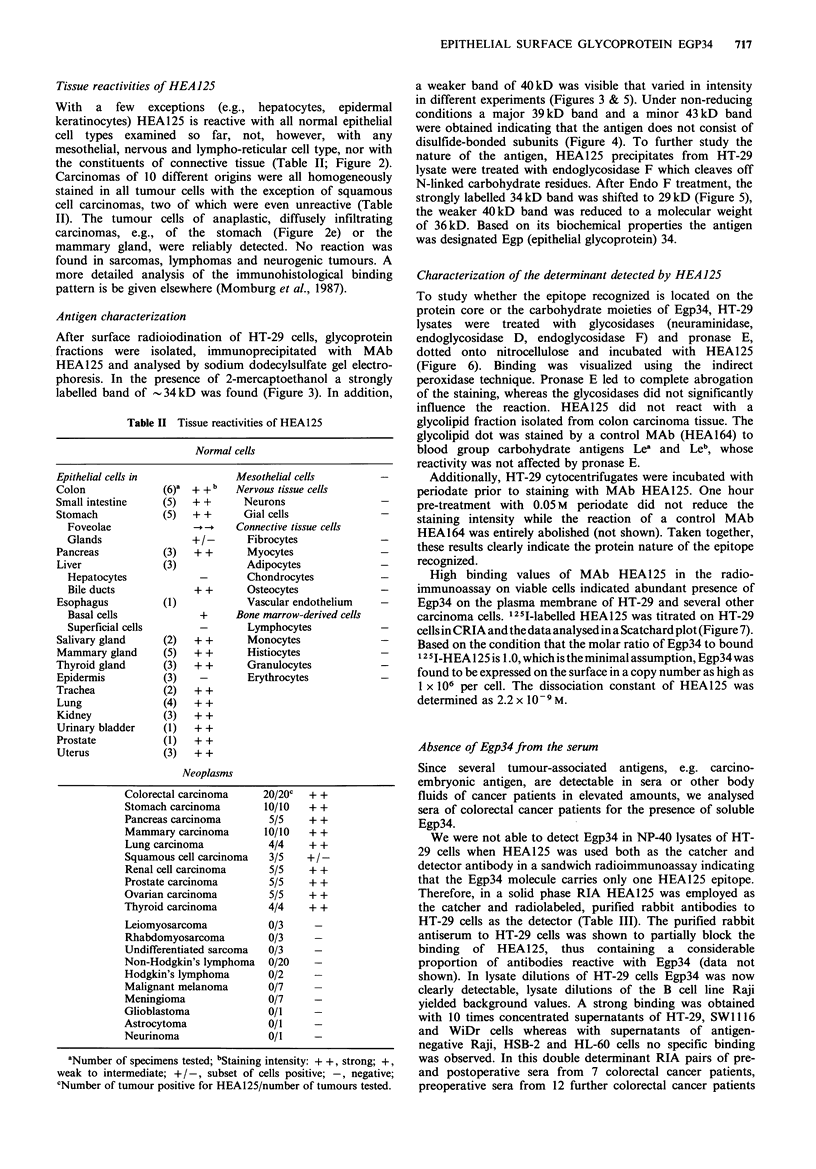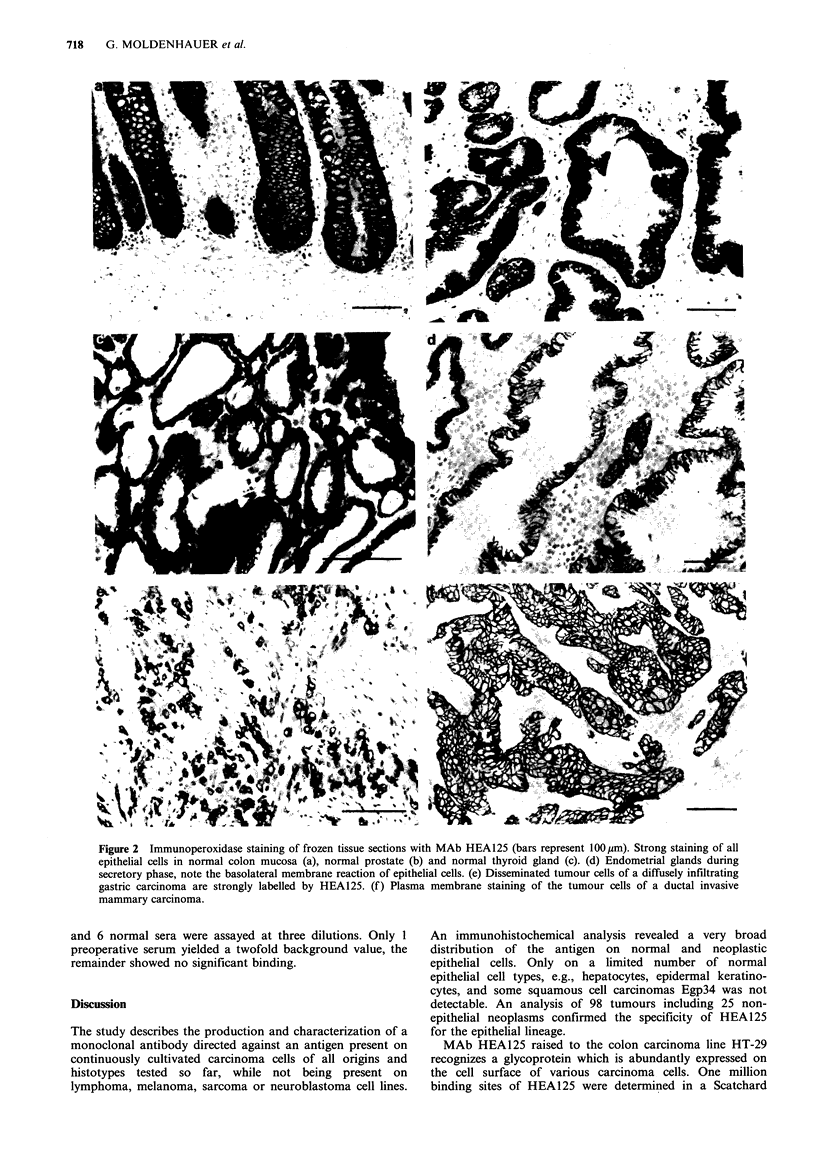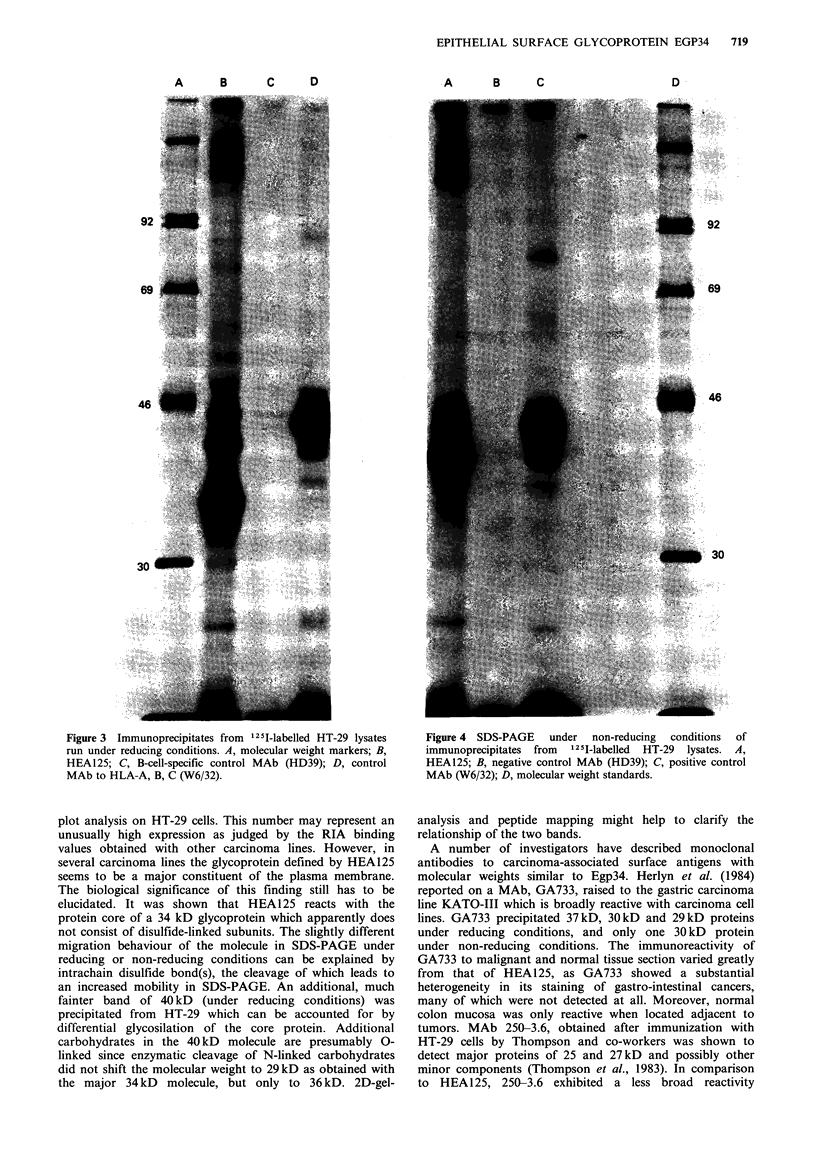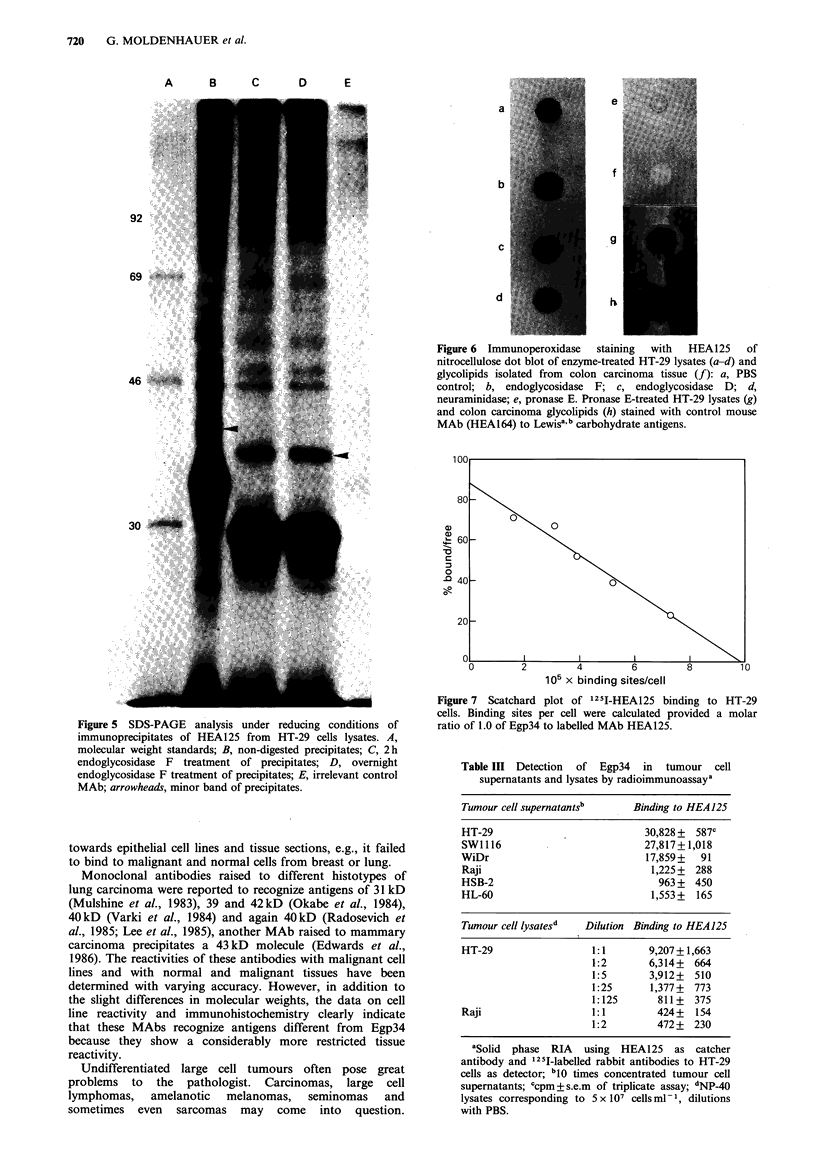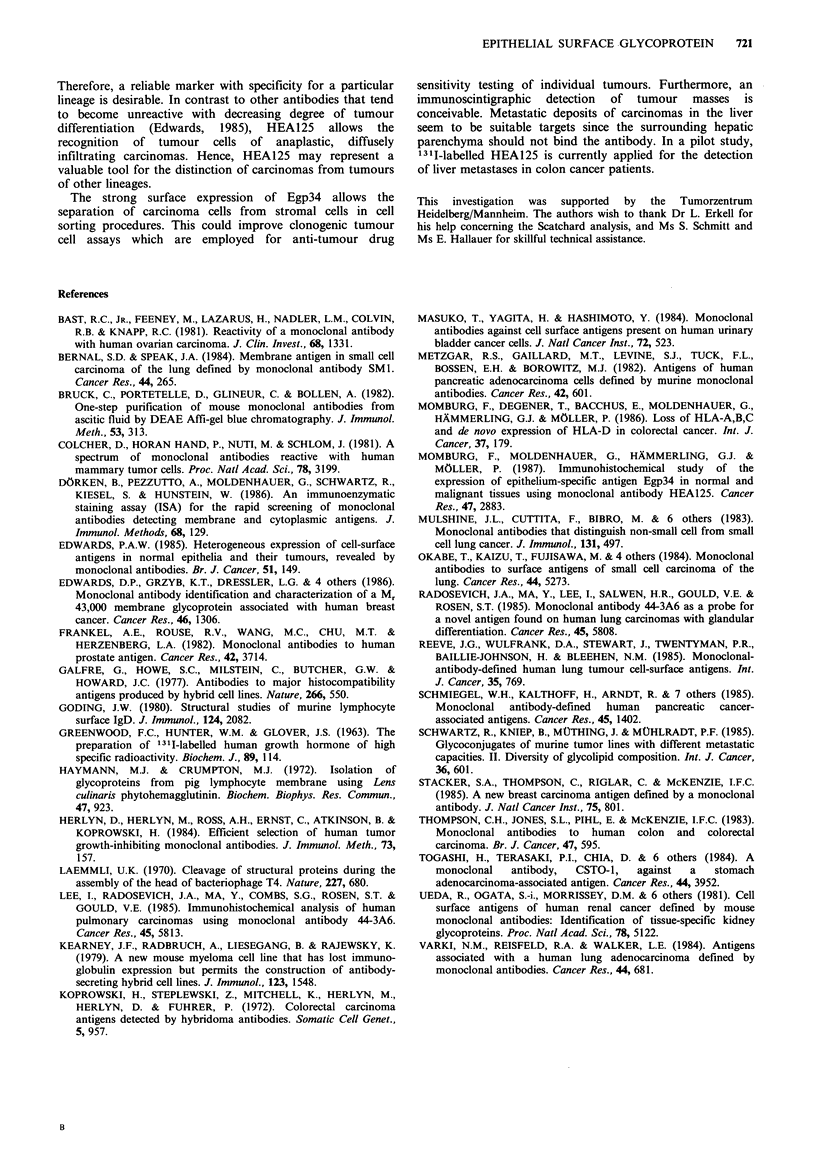# Epithelium-specific surface glycoprotein of Mr 34,000 is a widely distributed human carcinoma marker.

**DOI:** 10.1038/bjc.1987.276

**Published:** 1987-12

**Authors:** G. Moldenhauer, F. Momburg, P. Möller, R. Schwartz, G. J. Hämmerling

**Affiliations:** Institute of Immunology and Genetics, German Cancer Research Centre, Heidelberg.

## Abstract

**Images:**


Br. J. Cancer (1987), 56, 714–721 © The Macmillan Press Ltd., 1987

### Epithelium-specific surface glycoprotein of $M_r$ 34,000 is a widely distributed human carcinoma marker

G. Moldenhauer[1], F. Momburg[2], P. Möller[2], R. Schwartz[1] & G.J. Hämmerling[1]

[1]*Institute of Immunology and Genetics, German Cancer Research Centre, D-6900 Heidelberg and* [2]*Institute of Pathology, University of Heidelberg, D-6900 Heidelberg, Federal Republic of Germany.*

**Summary** An epthelial cell surface antigen is described which is defined by monoclonal antibody HEA125 (IgG1). The antibody was raised against the colon carcinoma cell line HT-29. Under reducing conditions HEA125 immunoprecipitates a surface glycoprotein of $M_r$ 34,000 which was designated Egp34. The antigen does not contain disulfide-linked subunits. A slightly different migration behavior under non-reducing conditions ($M_r$ 39,000) may be due to intrachain disulfide bonds. After enzymatic cleavage of N-linked carbohydrate residues the apparent molecular weight of the antigen was 29,000. Egp34 is a major cell surface component of HT-29 cells ($10^6$ molecules per cell). No antigen could be detected in the sera of colorectal cancer patients. A panel of malignant cell lines and normal cells was studied for surface expression of the antigen. 17/17 carcinoma lines of 6 different origins expressed the antigen, whereas 16/16 melanoma, neuroblastoma, sarcoma and lymphoma/leukaemia were unreactive as it was the case for normal fibroblasts and blood cells. Immunoperoxidase staining of frozen tissue sections with HEA125 demonstrated the presence of Egp34 in almost all normal epithelia and tumours derived therefrom. No reactivity with non-epithelial tissues was observed. Undifferentiated carcinomas of various origins homogeneously expressed Egp34. Therefore, HEA125 may become a valuable tool for the immunohistochemical diagnosis of carcinoma.

Since the advent of hybridoma technology numerous efforts have been made to generate monoclonal antibodies with specificity for plasma membrane antigens of selelcted epithelial cell types and their tumours (Koprowski *et al.*, 1972; Metzgar *et al.*, 1982; Schmiegel *et al.*, 1985; Togashi *et al.*, 1984; Colcher *et al.*, 1981; Stacker *et al.*, 1985; Bernal *et al.*, 1984; Reeve *et al.*, 1985; Bast *et al.*, 1981; Ueda *et al.*, 1981; Masuko *et al.*, 1984; Frankel *et al.*, 1982). Most monoclonal antibodies (MAbs) to epithelial cell surface components described so far recognize differentiation antigens that i) are not specific for the malignant phenotype, that ii) bind to the tumour cells of an individual carcinoma with considerable heterogeneity, and that iii) are undetectable in a subset of tumours of a given origin (reviewed in Edwards, 1985).

In contrast to antigens with a restricted and heterogeneous expression among human epithelial tumours, surface antigens with wide distribution and homogeneous expression in these neoplasms would be helpful in the diagnosis of anaplastic carcinomas in immunocytochemistry and immunohistochemistry provided the antigens are expressed in a strictly epithelium-specific manner. Antibodies to such antigens would be suitable tools for staining and targeting of viable carcinoma cells *in vitro* and *in vivo*. In the present investigation we report on a monoclonal antibody that reacts with a cell surface antigen of $M_r$ 34,000 present on all carcinoma cell lines tested, however, not on various cell lines and normal cells of non-epithelial origin. Biochemical characteristics of the antigen are described and the presence of soluble antigen in sera of cancer patients is examined. An immunohistological survey of human tissues demonstrates the strong and homogeneous expression of the antigen in most normal epithelial cell types and in carcinomas. An epithelium-specific pattern of expression of the $M_r$ 34,000 antigen is also found in tissues.

### Materials and methods

### *Cell lines and tissues*

Cell lines of various tissue origins as indicated in Table I were provided by the following institutions. From the American Type Culture Collection (Rockville, MD): WiDr, SW1116, HeLa, T-24, Raji, Daudi, P3-HR1, HL-60, U-937 and K-562; from Drs J. Fogh and M. Pfreundschuh (Memorial Sloan-Kettering Cancer Center, New York): HT-29, SK-LU-1, SK-LC-LL, SK-MES-1, Calu-1, SK-N-SH, HEp-2, ME-180, AlAb, P5A and F136-35-36. Several other colleagues kindly donated their cell lines: ChaGo (Dr K. Bosslet, Marburg, FRG); NCI-N417, SCLC-16H, SCLC-22M (Dr G. Bepler, Marburg, FRG); SW210-5 (Dr F. Herrmann, Boston, MA); MML-I, MML-III, MeWo (Dr W. Tilgen, Heidelberg, FRG); JM-1, Jurkat, HSB-2 (Dr B. Dörken, Heidelberg, FRG); LICR-LON-HMy-2 (Ludwig Institute of Cancer Research, UK).

Suspension cell lines were grown in RPMI 1640 medium completed with 10% heat-inactivated foetal calf serum, 2mM L-glutamine, 1mM pyruvate, penicillin-streptomycin $100\,U\,ml^{-1}$ (all components from Gibco, Karlsruhe, FRG). Adherent cell lines were cultivated in complete RPMI 1640 supplemented with $50\,\mu M$ 2-mercaptoethanol, 1% (v/v) non-essential amino acids (Seromed, Berlin, FRG) and $4\,IU\,l^{-1}$ human insulin (Novo, Mainz, FRG). Cell lines were mechanically harvested from monolayer cell cultures after treatment with 0.25% EDTA, washed twice in RPMI 1640 and used for cell binding assays and immunizations. P3X63Ag8.653 murine myeloma cells (Kearney *et al.*, 1979) were maintained in complete RPMI 1640 medium.

Specimens of normal and neoplastic tissues were obtained from surgical material and snap-frozen in liquid nitrogen within two hours after removal.

### *Immunization and hybridoma production*

Female BALB/c mice were immunized by i.p. injection of $\sim 2 \times 10^7$ viable HT-29 cells. The mice were boosted four times at four-week intervals with $1\text{–}2 \times 10^7$ HT-29 cells i.p., the last injection 3 days prior to the fusion experiment.

Fusions were performed essentially as described by Galfre *et al.* (1977). Splenocytes from immune spleens and Ag8.653 myeloma cells were mixed at a cell ratio of 5:1 and fused in 1.5ml of 45% polyethylene glycol 4000 (Merck, Darmstadt, FRG) in Dulbecco's PBS (Seromed) with 5% dimethylsulfoxide (Merck). After washing in Dulbecco's PBS, cells were resuspended in warm HAT medium (RPMI 1640 medium containing $13.611\,mg\,l^{-1}$ hypoxanthine, $0.176\,mg\,l^{-1}$ aminopterin and $3.876\,mg\,l^{-1}$ thymidine). Cells were

Correspondence: G. Moldenhauer.
Received 24 February 1987; and in revised form, 10 July 1987.

distributed in 24-well microtiter plates (Costar, Cambridge, MA) at $5 \times 10^5$ cells per well after preincubation for 48 h with $3 \times 10^4$ non-immune BALB/c peritoneal macrophages. Hybrids were kept in HAT selection medium for 2 weeks. On days 12 to 14 after fusion, supernatants from wells with visible colonies were assayed for cell binding in the immunoenzymatic staining assay. Hybridomas of interest were subcloned by limiting dilution on a feeder layer of BALB/c spleen cells ($5 \times 10^5$ cells $ml^{-1}$).

*Immunoenzymatic staining assay on fixed cells (ISA)*

The test was essentially performed as described by Dörken *et al.* (1986). Tissue culture cells in serum-free medium were distributed in Terasaki microtiter plates (Falcon, Oxnard, CA) at $3.3 \times 10^3$ cells per well pretreated overnight with poly-L-lysine (100 $\mu g\,ml^{-1}$ in PBS; Sigma, St. Louis, MO). Cells were fixed in 0.025% glutardialdehyde (Merck) for 10 min at room temperature. Plates were washed with PBS, blocked with PBS/0.2% gelatine (Merck)/0.1% $NaN_3$ and stored at 4°C.

For the immunostaining, cells were incubated with hybridoma supernatants (5 $\mu$l/well) for 1 h at room temperature. After 3 washings with PBS/gelatine, biotinylated goat anti-mouse Ig (Tago, Burlingame, CA) diluted 1:40 in PBS/5% pooled human IgG (Behringwerke) and avidin-peroxidase (Vector, Burlingame, CA) diluted 1:800 were added for 30 min each. Alternatively, a 4-step peroxidase anti-peroxidase technique was used as described previously (Momburg *et al.*, 1986). After 4 washings the substrate solution containing 0.4 mg $ml^{-1}$ 3-amino-9-ethylcarbazole (Sigma), 5% dimethylformamide (Sigma) and 0.015% $H_2O_2$ in 0.1 M acetate buffer (pH 5.2) was added for 10 min. The reaction (mostly ring-like stainings) was evaluated under a light microscope and scored −, +, ++, +++ in comparison to appropriate negative and positive controls.

*Immunoperoxidase staining of cytocentrifugates and tissue sections*

HT-29 cells in culture medium were cytocentrifuged onto glass slides, air-dried and fixed with acetone for 10 min. The cells were incubated with 0.005 M, 0.01 M and 0.05 M $NaIO_4$ (Merck) in PBS for 60 min at room temperature prior to staining with HEA125 as described below.

Frozen sections of 5 $\mu$m thickness were thoroughly air-dried and then acetone-fixed at room temperature for 10 min. After rehydration with PBS the sections were incubated for 60 min with purified HEA125 at a concentration of 10 $\mu g\,ml^{-1}$. Subsequently, sections were overlaid with rabbit anti-mouse Ig (produced by us) goat anti-rabbit IgG (Tago) and rabbit peroxidase anti-peroxidase complex (Dakopatts, Copenhagen, Denmark), each for 30 min at room temperature. The sandwich reagents were used at concentrations of 10 $\mu g\,ml^{-1}$, 20 $\mu g\,ml^{-1}$ and 1:50, respectively. The peroxidase substrate solution (see above, 'ISA') was added for 10 min. The sections were counterstained with Harris' hematoxylin and mounted with glycerol gelatine.

*Immunoprecipitation*

Viable HT-29 cells were radioiodinated by the lactoperoxidase method essentially as described by Goding (1980). Usually, $10^7$ cells (viability >95%) were radiolabelled with 0.5 mCi $^{125}I$ (Amersham Buchler) and subsequently solubilized in TBS (10 mM Tris, 150 mM NaCl, pH 7.4) containing 1% Nonidet P-40 (NP-40; Fluka), 1 mM phenylmethylsulphonylfluoride (Sigma) and 0.03 TIU $ml^{-1}$ aprotinin (Sigma). The glycoprotein fraction was isolated by a Lens culinaris lectin Sepharose-4B column (Pharmacia, Uppsala, Sweden) (Haymann & Crumpton, 1972). Purified glycoproteins were non-specifically precipitated by incubation with Protein A-Sepharose 4B (Pharmacia). For immunoprecipitation, $\sim 10^6$ cpm of glycoproteins were mixed with 10 $\mu$g purified MAb, 15 $\mu$g affinity-purified rabbit anti-mouse Ig and 20 $\mu$l Protein A-Sepharose 4B (50% solution). After overnight incubation at 4°C with gentle rotation, the adsorbent was washed and then boiled in reducing or non-reducing sodium dodecylsulfate (SDS) sample buffer. Samples were subjected to polyacrylamide slab gel electrophoresis (SDS-PAGE) using a discontinuous buffer system according to Laemmli (1970). Dried gels were visualized by exposure to Kodak X-Omat XR-5 X-ray film employing Cronex Lightning Plus intensifying screens. $^{14}C$-methylated molecular weight markers were obtained from Amersham Buchler (lysozyme, $M_r$ 14,300; carbonic anhydrase, $M_r$ 30,000; ovalbumin, $M_r$ 46,000; bovine serum albumin, $M_r$ 69,000; phosphorylase b, $M_r$ 92,500; myosin, $M_r$ 200,000).

For endo F treatment the immunoprecipitate was boiled 5 min in 30 $\mu$l 100 mM Tris/HCl, pH 8.0, containing 0.5% SDS, 1% 2-mercaptoethanol and 10 mM EDTA. After centrifugation 3 $\mu$l 10% NP-40 and 2 $\mu$l endoglycosidase F (0.048 U/ul; Boehringer, Mannheim, FRG) were added to the supernatant. Incubation was for 2 h at 37°C or overnight at room temperature, respectively. Endo F digests were analysed by SDS-PAGE in parallel with non-digested controls.

*Radiobinding assays*

HEA125 was purified from ascites fluid by DEAE Affi-Gel Blue (Biorad, Munic, FRG) chromatography (Bruck *et al.*, 1982). Radioiodination of MAb was performed by a slight modification of the chloramine-T method (Greenwood *et al.*, 1963) using 1 mCi $^{125}I$-iodide (Amersham Buchler, Braunschweig, FRG) per 100 $\mu$g of purified MAb.

The radioimmunoassay on live cells (CRIA) was performed in flexible PVC microtiter plates (Dynatech, Plochingen, FRG). Tissue culture cells were diluted in PBS/0.2% gelatine/5% pooled human IgG. 50 $\mu$l/well of target cell suspension containing $10^6$ viable cells were mixed with $^{125}I$-labelled HEA125 ($2 \times 10^6$ cpm in 100 $\mu$l) and allowed to react for 1 h at room temperature. After 3 washings the dried plate was sliced and the radioactivity of each well was determined in a gamma-counter. $^{125}I$-labelled irrelevant mouse monoclonal antibody of IgG1 subtype served as a negative control.

For quantitative determination of antigen sites per cell HT-29 cells were incubated with increasing amounts of $^{125}I$-HEA125 in triplicates. MAb binding to target cells was analysed in a Scatchard plot.

A double determinant RIA was performed with purified HEA125 coated to the wells of an immunoassay plate (2 $\mu g\,ml^{-1}$ antibody in 0.05 M sodium carbonate buffer, pH 9.6, overnight at 4°C). Unspecific binding sites were blocked with PBS/gelatine. Dried wells were incubated with serum dilutions (1:2, 1:5, 1:10 in PBS/Tween), tumour cell supernatants or tumour cell lysates overnight. After extensive washing with PBS/Tween $^{125}I$-labelled rabbit antiserum to HT-29 cells was added for 1 h. The rabbit antiserum reactive with Egp34 was purified by Protein A-Sepharose 4B (Pharmacia) prior to radioiodination by the chloramine-T method.

*Dot blot of enzyme-treated lysates*

HT-29 cells were lysed in extraction buffer ($10^7$ cells per 100 $\mu$l of 50 mM Tris/HCl, pH 7.5, with 5 mM iodoacetamide (Serva, Heidelberg, FRG), 2 mM EDTA, 1% NP-40) in the presence of proteinase inhibitors (see above) for all enzyme treatments except pronase. Lysates ($1.5 \times 10^7$ cells per enzyme) were incubated overnight at 37°C with equal volumes containing the following enzymes or PBS for control: endoglycosidase F (0.25 U in PBS + 5% n-octyl-L-glucoside (Sigma); endoglycosidase D (Boehringer), 0.012 U in

PBS + 5% n-octyl-L-glucoside; neuraminidase (Behringwerke, Marburg, FRG), 0.1 U in PBS; pronase E (Serva), 1 mg $100\,\mu l^{-1}$ PBS. After brief boiling of the mixture, $20\,\mu l$ ($= 10^6$ cells) were dotted onto nitrocellulose paper (Schleicher & Schüll, Dassel, FRG). Additionally, glycolipids isolated from colon carcinoma tissue by chloroform/methanol extraction (Schwartz *et al.*, 1985) were dotted. Unspecific binding sites were blocked with PBS/2% BSA for 1 h. Filters were subsequently incubated with purified HEA125 ($10\,\mu g\,ml^{-1}$) and peroxidase-conjugated goat anti-mouse IgG + IgM antiserum diluted 1:1000 (Jackson, Avondale, PA). Washings were done with PBS/Tween. The binding of antibody was visualized with diaminobenzidine (Fluka, Ulm, FRG) used at $1\,mg\,ml^{-1}$ in 0.05 M Tris/HCl (pH 7.6) with 0.01% $H_2O_2$.

### Results

### *Hybridoma production and initial screening*

Monoclonal antibody HEA125 was selected out of 960 hybridoma cultures obtained in 4 fusions following immunization with the human colon carcinoma cell line HT-29. After testing for mouse immunoglobulin secretion by ELISA the culture supernatants were initially screened in the ISA on fixed cells. Twenty-eight hybridomas that recognized each of the carcinoma lines HT-29, SK-LU-1 and HeLa but neither the melanoma line MML-I, nor the lymphoma lines Raji and JM-1 were cloned by limiting dilution and further characterized. MAb HEA125 was determined to be of the IgG1, K isotype.

### *Reactivity with tumour cell lines and normal blood cells*

Purified MAb HEA125 was screened on a panel of human malignant cell lines, normal fibroblasts and peripheral blood cells using the CRIA on viable cells or the ISA on fixed cells as indicated in Table I. HEA125 bound to all carcinoma cell lines tested, irrespective of the use of unfixed or glutaraldehyde-fixed cells. A particularly strong reaction was obtained with colon carcinoma and small cell lung carcinoma cell lines. HEA125 did not significantly discriminate between different histotypes of carcinoma, nor did it show organ specificity. Figure 1 illustrates the strong membrane staining of fixed ME-180 cervix carcinoma cells by HEA125. By contrast, no reaction was found with any of the non-carcinoma cell lines used, which were derived from melanoma, neuroblastoma, sarcoma and leukaemia/lymphoma of the B, T and myelomonocytic lineage. Accordingly, no binding was found to normal lung fibroblasts, as it was the case for the cell types of normal peripheral blood.

**Figure 1** Immunoperoxidase staining of glutardialdehyde-fixed ME-180 cervix carcinoma cells with MAb HEA125. The strong reaction with the cell surface results in ring-like stainings (×962).

**Table I** Binding of MAb HEA125 to human malignant cell lines and normal blood cells in radioimmunoassay on live cells and immunoenzymatic staining assay on fixed cells

| | *CRIA* | *ISA* | | *CRIA* | *ISA* |
|---|---|---|---|---|---|
| *Colon carcinoma* | | | *Melanoma* | | |
| HT-29 | ++++ | +++ | MML-1 | | – |
| WiDr | | +++ | MML-3 | | – |
| SW1116 | | +++ | Mewo | | – |
| *Lung carcinoma* | | | *Neuroblastoma* | | |
| adenocarcinoma | | | SK-N-SH | | – |
| SK-LU-1 | +++ | ++ | *Sarcoma* | | |
| squamous cell ca. | | | P5A | | – |
| SK-LC-LL | ++ | ++ | *B cell lymphoma* | | |
| SK-MES-1 | | + | Raji | – | |
| Calu-1 | | + | Daudi | – | |
| large cell ca. | | | P3-HR1 | – | |
| ChaGo | ++ | ++ | LICR-LON-HMy-2 | – | |
| small cell ca. | | | *T cell lymphoma* | | |
| NCI-N417 | ++++ | | JM-1 | – | |
| SCLC-16H | ++++ | | Jurkat | – | |
| SCLC-22H | +++ | | HSB-2 | – | |
| SW210-5 | ++++ | | Molt-3 | – | |
| *Breast carcinoma* | | | *Myelocytic leukemia* | | |
| AlAb | | +++ | HL-60 | – | |
| *Cervix carcinoma* | | | U-937 | – | |
| HeLa | ++ | | K-562 | – | |
| ME-180 | | +++ | *Normal foetal fibroblasts* | | |
| *Larynx carcinoma* | | | F136-35-36 | | – |
| HEp-2 | +++ | ++ | *Normal blood cells* | | |
| *Bladder carcinoma* | | | lymphocytes | | – |
| T-24 | | + | monocytes | | – |
| | | | granulocytes | | – |
| | | | erythrocytes A, B, 0 | | – |

[a]CRIA reactions were scored –, 0–3 × control value obtained with irrelevant IgG1 MAb (c.v.); +, 3–10 × c.v.; ++, 10–30 × c.v.; +++, 30–100 × c.v.; ++++, > 100 × c.v.; [b]ISA stainings were scored –, negative; +, weak; ++, intermediate; +++, strong.

*Tissue reactivities of HEA125*

With a few exceptions (e.g., hepatocytes, epidermal keratinocytes) HEA125 is reactive with all normal epithelial cell types examined so far, not, however, with any mesothelial, nervous and lympho-reticular cell type, nor with the constituents of connective tissue (Table II; Figure 2). Carcinomas of 10 different origins were all homogeneously stained in all tumour cells with the exception of squamous cell carcinomas, two of which were even unreactive (Table II). The tumour cells of anaplastic, diffusely infiltrating carcinomas, e.g., of the stomach (Figure 2e) or the mammary gland, were reliably detected. No reaction was found in sarcomas, lymphomas and neurogenic tumours. A more detailed analysis of the immunohistological binding pattern is be given elsewhere (Momburg *et al.*, 1987).

*Antigen characterization*

After surface radioiodination of HT-29 cells, glycoprotein fractions were isolated, immunoprecipitated with MAb HEA125 and analysed by sodium dodecylsulfate gel electrophoresis. In the presence of 2-mercaptoethanol a strongly labelled band of ~34 kD was found (Figure 3). In addition, a weaker band of 40 kD was visible that varied in intensity in different experiments (Figures 3 & 5). Under non-reducing conditions a major 39 kD band and a minor 43 kD band were obtained indicating that the antigen does not consist of disulfide-bonded subunits (Figure 4). To further study the nature of the antigen, HEA125 precipitates from HT-29 lysate were treated with endoglycosidase F which cleaves off N-linked carbohydrate residues. After Endo F treatment, the strongly labelled 34 kD band was shifted to 29 kD (Figure 5), the weaker 40 kD band was reduced to a molecular weight of 36 kD. Based on its biochemical properties the antigen was designated Egp (epithelial glycoprotein) 34.

**Table II** Tissue reactivities of HEA125

| *Normal cells* | | | | |
|---|---|---|---|---|
| *Epithelial cells in* | | | *Mesothelial cells* | − |
| Colon | (6)[a] | + +[b] | *Nervous tissue cells* | |
| Small intestine | (5) | + + | Neurons | − |
| Stomach | (5) | + + | Gial cells | − |
| Foveolae | | →→ | *Connective tissue cells* | |
| Glands | | +/− | Fibrocytes | − |
| Pancreas | (3) | + + | Myocytes | − |
| Liver | (3) | | Adipocytes | − |
| Hepatocytes | | − | Chondrocytes | − |
| Bile ducts | | + + | Osteocytes | − |
| Esophagus | (1) | | Vascular endothelium | − |
| Basal cells | | + | *Bone marrow-derived cells* | |
| Superficial cells | | − | Lymphocytes | − |
| Salivary gland | (2) | + + | Monocytes | − |
| Mammary gland | (5) | + + | Histiocytes | − |
| Thyroid gland | (3) | + + | Granulocytes | − |
| Epidermis | (3) | − | Erythrocytes | − |
| Trachea | (2) | + + | | |
| Lung | (4) | + + | | |
| Kidney | (3) | + + | | |
| Urinary bladder | (1) | + + | | |
| Prostate | (1) | + + | | |
| Uterus | (3) | + + | | |

| *Neoplasms* | | |
|---|---|---|
| Colorectal carcinoma | 20/20[c] | + + |
| Stomach carcinoma | 10/10 | + + |
| Pancreas carcinoma | 5/5 | + + |
| Mammary carcinoma | 10/10 | + + |
| Lung carcinoma | 4/4 | + + |
| Squamous cell carcinoma | 3/5 | +/− |
| Renal cell carcinoma | 5/5 | + + |
| Prostate carcinoma | 5/5 | + + |
| Ovarian carcinoma | 5/5 | + + |
| Thyroid carcinoma | 4/4 | + + |
| Leiomyosarcoma | 0/3 | − |
| Rhabdomyosarcoma | 0/3 | − |
| Undifferentiated sarcoma | 0/3 | − |
| Non-Hodgkin's lymphoma | 0/20 | − |
| Hodgkin's lymphoma | 0/2 | − |
| Malignant melanoma | 0/7 | − |
| Meningioma | 0/7 | − |
| Glioblastoma | 0/1 | − |
| Astrocytoma | 0/1 | − |
| Neurinoma | 0/1 | − |

[a]Number of specimens tested; [b]Staining intensity: + +, strong; +, weak to intermediate; +/−, subset of cells positive; −, negative; [c]Number of tumour positive for HEA125/number of tumours tested.

*Characterization of the determinant detected by HEA125*

To study whether the epitope recognized is located on the protein core or the carbohydrate moieties of Egp34, HT-29 lysates were treated with glycosidases (neuraminidase, endoglycosidase D, endoglycosidase F) and pronase E, dotted onto nitrocellulose and incubated with HEA125 (Figure 6). Binding was visualized using the indirect peroxidase technique. Pronase E led to complete abrogation of the staining, whereas the glycosidases did not significantly influence the reaction. HEA125 did not react with a glycolipid fraction isolated from colon carcinoma tissue. The glycolipid dot was stained by a control MAb (HEA164) to blood group carbohydrate antigens $Le^a$ and $Le^b$, whose reactivity was not affected by pronase E.

Additionally, HT-29 cytocentrifugates were incubated with periodate prior to staining with MAb HEA125. One hour pre-treatment with 0.05 M periodate did not reduce the staining intensity while the reaction of a control MAb HEA164 was entirely abolished (not shown). Taken together, these results clearly indicate the protein nature of the epitope recognized.

High binding values of MAb HEA125 in the radioimmunoassay on viable cells indicated abundant presence of Egp34 on the plasma membrane of HT-29 and several other carcinoma cells. $^{125}$I-labelled HEA125 was titrated on HT-29 cells in CRIA and the data analysed in a Scatchard plot (Figure 7). Based on the condition that the molar ratio of Egp34 to bound $^{125}$I-HEA125 is 1.0, which is the minimal assumption, Egp34 was found to be expressed on the surface in a copy number as high as $1 \times 10^6$ per cell. The dissociation constant of HEA125 was determined as $2.2 \times 10^{-9}$ M.

*Absence of Egp34 from the serum*

Since several tumour-associated antigens, e.g. carcinoembryonic antigen, are detectable in sera or other body fluids of cancer patients in elevated amounts, we analysed sera of colorectal cancer patients for the presence of soluble Egp34.

We were not able to detect Egp34 in NP-40 lysates of HT-29 cells when HEA125 was used both as the catcher and detector antibody in a sandwich radioimmunoassay indicating that the Egp34 molecule carries only one HEA125 epitope. Therefore, in a solid phase RIA HEA125 was employed as the catcher and radiolabeled, purified rabbit antibodies to HT-29 cells as the detector (Table III). The purified rabbit antiserum to HT-29 cells was shown to partially block the binding of HEA125, thus containing a considerable proportion of antibodies reactive with Egp34 (data not shown). In lysate dilutions of HT-29 cells Egp34 was now clearly detectable, lysate dilutions of the B cell line Raji yielded background values. A strong binding was obtained with 10 times concentrated supernatants of HT-29, SW1116 and WiDr cells whereas with supernatants of antigen-negative Raji, HSB-2 and HL-60 cells no specific binding was observed. In this double determinant RIA pairs of pre- and postoperative sera from 7 colorectal cancer patients, preoperative sera from 12 further colorectal cancer patients

**Figure 2** Immunoperoxidase staining of frozen tissue sections with MAb HEA125 (bars represent 100 $\mu$m). Strong staining of all epithelial cells in normal colon mucosa (a), normal prostate (b) and normal thyroid gland (c). (d) Endometrial glands during secretory phase, note the basolateral membrane reaction of epithelial cells. (e) Disseminated tumour cells of a diffusely infiltrating gastric carcinoma are strongly labelled by HEA125. (f) Plasma membrane staining of the tumour cells of a ductal invasive mammary carcinoma.

and 6 normal sera were assayed at three dilutions. Only 1 preoperative serum yielded a twofold background value, the remainder showed no significant binding.

### Discussion

The study describes the production and characterization of a monoclonal antibody directed against an antigen present on continuously cultivated carcinoma cells of all origins and histotypes tested so far, while not being present on lymphoma, melanoma, sarcoma or neuroblastoma cell lines. An immunohistochemical analysis revealed a very broad distribution of the antigen on normal and neoplastic epithelial cells. Only on a limited number of normal epithelial cell types, e.g., hepatocytes, epidermal keratinocytes, and some squamous cell carcinomas Egp34 was not detectable. An analysis of 98 tumours including 25 non-epithelial neoplasms confirmed the specificity of HEA125 for the epithelial lineage.

MAb HEA125 raised to the colon carcinoma line HT-29 recognizes a glycoprotein which is abundantly expressed on the cell surface of various carcinoma cells. One million binding sites of HEA125 were determined in a Scatchard

Figure 3 Immunoprecipitates from $^{125}$I-labelled HT-29 lysates run under reducing conditions. *A*, molecular weight markers; *B*, HEA125; *C*, B-cell-specific control MAb (HD39); *D*, control MAb to HLA-A, B, C (W6/32).

**Figure 4** SDS-PAGE under non-reducing conditions of immunoprecipitates from $^{125}$I-labelled HT-29 lysates. *A*, HEA125; *B*, negative control MAb (HD39); *C*, positive control MAb (W6/32); *D*, molecular weight standards.

plot analysis on HT-29 cells. This number may represent an unusually high expression as judged by the RIA binding values obtained with other carcinoma lines. However, in several carcinoma lines the glycoprotein defined by HEA125 seems to be a major constituent of the plasma membrane. The biological significance of this finding still has to be elucidated. It was shown that HEA125 reacts with the protein core of a 34 kD glycoprotein which apparently does not consist of disulfide-linked subunits. The slightly different migration behaviour of the molecule in SDS-PAGE under reducing or non-reducing conditions can be explained by intrachain disulfide bond(s), the cleavage of which leads to an increased mobility in SDS-PAGE. An additional, much fainter band of 40 kD (under reducing conditions) was precipitated from HT-29 which can be accounted for by differential glycosilation of the core protein. Additional carbohydrates in the 40 kD molecule are presumably O-linked since enzymatic cleavage of N-linked carbohydrates did not shift the molecular weight to 29 kD as obtained with the major 34 kD molecule, but only to 36 kD. 2D-gel-analysis and peptide mapping might help to clarify the relationship of the two bands.

A number of investigators have described monoclonal antibodies to carcinoma-associated surface antigens with molecular weights similar to Egp34. Herlyn *et al.* (1984) reported on a MAb, GA733, raised to the gastric carcinoma line KATO-III which is broadly reactive with carcinoma cell lines. GA733 precipitated 37 kD, 30 kD and 29 kD proteins under reducing conditions, and only one 30 kD protein under non-reducing conditions. The immunoreactivity of GA733 to malignant and normal tissue section varied greatly from that of HEA125, as GA733 showed a substantial heterogeneity in its staining of gastro-intestinal cancers, many of which were not detected at all. Moreover, normal colon mucosa was only reactive when located adjacent to tumors. MAb 250–3.6, obtained after immunization with HT-29 cells by Thompson and co-workers was shown to detect major proteins of 25 and 27 kD and possibly other minor components (Thompson *et al.*, 1983). In comparison to HEA125, 250–3.6 exhibited a less broad reactivity

**Figure 5** SDS-PAGE analysis under reducing conditions of immunoprecipitates of HEA125 from HT-29 cells lysates. *A*, molecular weight standards; *B*, non-digested precipitates; *C*, 2 h endoglycosidase F treatment of precipitates; *D*, overnight endoglycosidase F treatment of precipitates; *E*, irrelevant control MAb; *arrowheads*, minor band of precipitates.

towards epithelial cell lines and tissue sections, e.g., it failed to bind to malignant and normal cells from breast or lung.

Monoclonal antibodies raised to different histotypes of lung carcinoma were reported to recognize antigens of 31 kD (Mulshine *et al.*, 1983), 39 and 42 kD (Okabe *et al.*, 1984), 40 kD (Varki *et al.*, 1984) and again 40 kD (Radosevich *et al.*, 1985; Lee *et al.*, 1985), another MAb raised to mammary carcinoma precipitates a 43 kD molecule (Edwards *et al.*, 1986). The reactivities of these antibodies with malignant cell lines and with normal and malignant tissues have been determined with varying accuracy. However, in addition to the slight differences in molecular weights, the data on cell line reactivity and immunohistochemistry clearly indicate that these MAbs recognize antigens different from Egp34 because they show a considerably more restricted tissue reactivity.

Undifferentiated large cell tumours often pose great problems to the pathologist. Carcinomas, large cell lymphomas, amelanotic melanomas, seminomas and sometimes even sarcomas may come into question.

**Figure 6** Immunoperoxidase staining with HEA125 of nitrocellulose dot blot of enzyme-treated HT-29 lysates (*a–d*) and glycolipids isolated from colon carcinoma tissue (*f*): *a*, PBS control; *b*, endoglycosidase F; *c*, endoglycosidase D; *d*, neuraminidase; *e*, pronase E. Pronase E-treated HT-29 lysates (*g*) and colon carcinoma glycolipids (*h*) stained with control mouse MAb (HEA164) to Lewis[a,b] carbohydrate antigens.

**Figure 7** Scatchard plot of $^{125}$I-HEA125 binding to HT-29 cells. Binding sites per cell were calculated provided a molar ratio of 1.0 of Egp34 to labelled MAb HEA125.

**Table III** Detection of Egp34 in tumour cell supernatants and lysates by radioimmunoassay[a]

| *Tumour cell supernatants*[b] | | *Binding to HEA125* |
|---|---|---|
| HT-29 | | 30,828 ± 587[c] |
| SW1116 | | 27,817 ± 1,018 |
| WiDr | | 17,859 ± 91 |
| Raji | | 1,225 ± 288 |
| HSB-2 | | 963 ± 450 |
| HL-60 | | 1,553 ± 165 |
| *Tumour cell lysates*[d] | *Dilution* | *Binding to HEA125* |
| HT-29 | 1:1 | 9,207 ± 1,663 |
| | 1:2 | 6,314 ± 664 |
| | 1:5 | 3,912 ± 510 |
| | 1:25 | 1,377 ± 773 |
| | 1:125 | 811 ± 375 |
| Raji | 1:1 | 424 ± 154 |
| | 1:2 | 472 ± 230 |

[a]Solid phase RIA using HEA125 as catcher antibody and $^{125}$I-labelled rabbit antibodies to HT-29 cells as detector; [b]10 times concentrated tumour cell supernatants; [c]cpm ± s.e.m of triplicate assay; [d]NP-40 lysates corresponding to $5 \times 10^7$ cells ml$^{-1}$, dilutions with PBS.

Therefore, a reliable marker with specificity for a particular lineage is desirable. In contrast to other antibodies that tend to become unreactive with decreasing degree of tumour differentiation (Edwards, 1985), HEA125 allows the recognition of tumour cells of anaplastic, diffusely infiltrating carcinomas. Hence, HEA125 may represent a valuable tool for the distinction of carcinomas from tumours of other lineages.

The strong surface expression of Egp34 allows the separation of carcinoma cells from stromal cells in cell sorting procedures. This could improve clonogenic tumour cell assays which are employed for anti-tumour drug sensitivity testing of individual tumours. Furthermore, an immunoscintigraphic detection of tumour masses is conceivable. Metastatic deposits of carcinomas in the liver seem to be suitable targets since the surrounding hepatic parenchyma should not bind the antibody. In a pilot study, $^{131}$I-labelled HEA125 is currently applied for the detection of liver metastases in colon cancer patients.

This investigation was supported by the Tumorzentrum Heidelberg/Mannheim. The authors wish to thank Dr L. Erkell for his help concerning the Scatchard analysis, and Ms S. Schmitt and Ms E. Hallauer for skillful technical assistance.

### References

BAST, R.C., JR., FEENEY, M., LAZARUS, H., NADLER, L.M., COLVIN, R.B. & KNAPP, R.C. (1981). Reactivity of a monoclonal antibody with human ovarian carcinoma. *J. Clin. Invest.*, **68**, 1331.

BERNAL, S.D. & SPEAK, J.A. (1984). Membrane antigen in small cell carcinoma of the lung defined by monoclonal antibody SM1. *Cancer Res.*, **44**, 265.

BRUCK, C., PORTETELLE, D., GLINEUR, C. & BOLLEN, A. (1982). One-step purification of mouse monoclonal antibodies from ascitic fluid by DEAE Affi-gel blue chromatography. *J. Immunol. Meth.*, **53**, 313.

COLCHER, D., HORAN HAND, P., NUTI, M. & SCHLOM, J. (1981). A spectrum of monoclonal antibodies reactive with human mammary tumor cells. *Proc. Natl Acad. Sci.*, **78**, 3199.

DÖRKEN, B., PEZZUTTO, A., MOLDENHAUER, G., SCHWARTZ, R., KIESEL, S. & HUNSTEIN, W. (1986). An immunoenzymatic staining assay (ISA) for the rapid screening of monoclonal antibodies detecting membrane and cytoplasmic antigens. *J. Immunol. Methods*, **68**, 129.

EDWARDS, P.A.W. (1985). Heterogeneous expression of cell-surface antigens in normal epithelia and their tumours, revealed by monoclonal antibodies. *Br. J. Cancer*, **51**, 149.

EDWARDS, D.P., GRZYB, K.T., DRESSLER, L.G. & 4 others (1986). Monoclonal antibody identification and characterization of a $M_r$ 43,000 membrane glycoprotein associated with human breast cancer. *Cancer Res.*, **46**, 1306.

FRANKEL, A.E., ROUSE, R.V., WANG, M.C., CHU, M.T. & HERZENBERG, L.A. (1982). Monoclonal antibodies to human prostate antigen. *Cancer Res.*, **42**, 3714.

GALFRE, G., HOWE, S.C., MILSTEIN, C., BUTCHER, G.W. & HOWARD, J.C. (1977). Antibodies to major histocompatibility antigens produced by hybrid cell lines. *Nature*, **266**, 550.

GODING, J.W. (1980). Structural studies of murine lymphocyte surface IgD. *J. Immunol.*, **124**, 2082.

GREENWOOD, F.C., HUNTER, W.M. & GLOVER, J.S. (1963). The preparation of $^{131}$I-labelled human growth hormone of high specific radioactivity. *Biochem. J.*, **89**, 114.

HAYMANN, M.J. & CRUMPTON, M.J. (1972). Isolation of glycoproteins from pig lymphocyte membrane using *Lens culinaris* phytohemagglutinin. *Biochem. Biophys. Res. Commun.*, **47**, 923.

HERLYN, D., HERLYN, M., ROSS, A.H., ERNST, C., ATKINSON, B. & KOPROWSKI, H. (1984). Efficient selection of human tumor growth-inhibiting monoclonal antibodies. *J. Immunol. Meth.*, **73**, 157.

LAEMMLI, U.K. (1970). Cleavage of structural proteins during the assembly of the head of bacteriophage T4. *Nature*, **227**, 680.

LEE, I., RADOSEVICH, J.A., MA, Y., COMBS, S.G., ROSEN, S.T. & GOULD, V.E. (1985). Immunohistochemical analysis of human pulmonary carcinomas using monoclonal antibody 44-3A6. *Cancer Res.*, **45**, 5813.

KEARNEY, J.F., RADBRUCH, A., LIESEGANG, B. & RAJEWSKY, K. (1979). A new mouse myeloma cell line that has lost immunoglobulin expression but permits the construction of antibody-secreting hybrid cell lines. *J. Immunol.*, **123**, 1548.

KOPROWSKI, H., STEPLEWSKI, Z., MITCHELL, K., HERLYN, M., HERLYN, D. & FUHRER, P. (1972). Colorectal carcinoma antigens detected by hybridoma antibodies. *Somatic Cell Genet.*, **5**, 957.

MASUKO, T., YAGITA, H. & HASHIMOTO, Y. (1984). Monoclonal antibodies against cell surface antigens present on human urinary bladder cancer cells. *J. Natl Cancer Inst.*, **72**, 523.

METZGAR, R.S., GAILLARD, M.T., LEVINE, S.J., TUCK, F.L., BOSSEN, E.H. & BOROWITZ, M.J. (1982). Antigens of human pancreatic adenocarcinoma cells defined by murine monoclonal antibodies. *Cancer Res.*, **42**, 601.

MOMBURG, F., DEGENER, T., BACCHUS, E., MOLDENHAUER, G., HÄMMERLING, G.J. & MÖLLER, P. (1986). Loss of HLA-A,B,C and *de novo* expression of HLA-D in colorectal cancer. *Int. J. Cancer*, **37**, 179.

MOMBURG, F., MOLDENHAUER, G., HÄMMERLING, G.J. & MÖLLER, P. (1987). Immunohistochemical study of the expression of epithelium-specific antigen Egp34 in normal and malignant tissues using monoclonal antibody HEA125. *Cancer Res.*, **47**, 2883.

MULSHINE, J.L., CUTTITA, F., BIBRO, M. & 6 others (1983). Monoclonal antibodies that distinguish non-small cell from small cell lung cancer. *J. Immunol.*, **131**, 497.

OKABE, T., KAIZU, T., FUJISAWA, M. & 4 others (1984). Monoclonal antibodies to surface antigens of small cell carcinoma of the lung. *Cancer Res.*, **44**, 5273.

RADOSEVICH, J.A., MA, Y., LEE, I., SALWEN, H.R., GOULD, V.E. & ROSEN, S.T. (1985). Monoclonal antibody 44-3A6 as a probe for a novel antigen found on human lung carcinomas with glandular differentiation. *Cancer Res.*, **45**, 5808.

REEVE, J.G., WULFRANK, D.A., STEWART, J., TWENTYMAN, P.R., BAILLIE-JOHNSON, H. & BLEEHEN, N.M. (1985). Monoclonal-antibody-defined human lung tumour cell-surface antigens. *Int. J. Cancer*, **35**, 769.

SCHMIEGEL, W.H., KALTHOFF, H., ARNDT, R. & 7 others (1985). Monoclonal antibody-defined human pancreatic cancer-associated antigens. *Cancer Res.*, **45**, 1402.

SCHWARTZ, R., KNIEP, B., MÜTHING, J. & MÜHLRADT, P.F. (1985). Glycoconjugates of murine tumor lines with different metastatic capacities. II. Diversity of glycolipid composition. *Int. J. Cancer*, **36**, 601.

STACKER, S.A., THOMPSON, C., RIGLAR, C. & MCKENZIE, I.F.C. (1985). A new breast carcinoma antigen defined by a monoclonal antibody. *J. Natl Cancer Inst.*, **75**, 801.

THOMPSON, C.H., JONES, S.L., PIHL, E. & MCKENZIE, I.F.C. (1983). Monoclonal antibodies to human colon and colorectal carcinoma. *Br. J. Cancer*, **47**, 595.

TOGASHI, H., TERASAKI, P.I., CHIA, D. & 6 others (1984). A monoclonal antibody, CSTO-1, against a stomach adenocarcinoma-associated antigen. *Cancer Res.*, **44**, 3952.

UEDA, R., OGATA, S.-I., MORRISSEY, D.M. & 6 others (1981). Cell surface antigens of human renal cancer defined by mouse monoclonal antibodies: Identification of tissue-specific kidney glycoproteins. *Proc. Natl Acad. Sci.*, **78**, 5122.

VARKI, N.M., REISFELD, R.A. & WALKER, L.E. (1984). Antigens associated with a human lung adenocarcinoma defined by monoclonal antibodies. *Cancer Res.*, **44**, 681.